# A retrospective cohort study of adverse events in patients undergoing orthopaedic surgery

**DOI:** 10.1186/s13037-017-0129-x

**Published:** 2017-05-11

**Authors:** Joel J. Gagnier, Hal Morgenstern, Patrick Kellam

**Affiliations:** 1 Department of Orthopaedic Surgery, University of Michigan, Ann Arbor, MI USA; 2 Department of Epidemiology, School of Public Health, University of Michigan, Ann Arbor, MI USA; 3 Department of Environmental Health Sciences, School of Public Health, University of Michigan, Ann Arbor, MI USA; 4 Department of Urology, Medical School, University of Michigan, Ann Arbor, MI USA; 5 School of Medicine, University of North Carolina, Chapel Hill, NC USA

**Keywords:** Adverse Events, Outcomes, Orthopaedic Surgery, Total Knee Arthroplasty, Total Hip Arthroplasty, Comorbidities

## Abstract

**Background:**

This study’s objective was to identify adverse events following common orthopaedic procedures, and to estimate the incidence rates and risks of these events and their associations with age, sex, and comorbidities.

**Methods:**

This retrospective cohort study manually reviewed and extracted electronic medical data on the incidence and predictors of adverse events that occurred within 90 days of the 50 most frequent orthopaedic surgeries at an academic hospital in 2010. We also extracted demographic data, baseline comorbidities, and duration of follow-up (≤90 days). Patients were scored on the Charlson Comorbidity Index (CCI) and the Functional Comorbidity Index (FCI). We estimated incidence rates and risks for all events and associations using regression methods. Prolonged pain 42-days post-surgery was treated as a separate outcome.

**Results:**

We included 1,552 patients; average age was 53.4 years, and 51.7% were female. A total of 1,148 adverse events were identified in 729 patients. The incidence rate of all adverse events was 10 events per 1,000 person-days at risk; 47% of all patients experienced at least one adverse event within 90 days. The most frequent events were prolonged pain (31% of all adverse events) and persistent swelling (7%). We found positive associations between both comorbidity scores and the incidence rate and 90-day risk of all adverse events, excluding pain, adjusting for age and sex (neither of which was associated with adverse events); the association was stronger for the FCI than for the CCI. For total hip arthroplasty (THA) and total knee arthroplasty (TKA), the incidence rate of all adverse events, excluding pain, was positively associated with both comorbidity scores and age; the 90-day risk was positively associate with the FCI score and male sex. The prevalence of prolonged pain at 42 days was greater in patients with higher FCI scores; for THA and TKA only, pain prevalence was greater in those with higher FCI scores and in men.

**Conclusions:**

Adverse events are frequent following common orthopaedic procedures. The incidence is greatest for patients with more functional comorbidities. For THA and TKA procedures, being male and being older are also associated with a greater incidence of adverse events.

## Background

A large number of adverse events (AE) are encountered during hospitalization, in particular during and after surgical procedures [1–5]. The operating room is a complex environment with a number of factors that increase the risk of AEs [6–12]. In particular, it contains a high concentration of information, can be very fast paced, has many patient transfers, and many person-machine interactions [6, 7]. A recent systematic review of the incidence and nature of in-hospital adverse events found that 58% of all events were related to surgery with general surgery and orthopaedic surgery being the largest contributors [10]. In addition, some research findings suggest that a large proportion of surgical AEs are preventable (e.g., [9, 10]). Consequently, several surgical safety mechanisms, including general surgery checklists, have been developed and tested across several surgical disciplines [13–16].

For example, the Surgical Safety Checklist (SSC) [13–16] and the SURgical Patient Safety System (SURPASS) checklist were developed [15, 16] and both appear to decrease the risk of complications. These results are promising but, these checklists do not specifically address unique characteristics of individual surgical specialties. Several researchers have suggested that surgical safety checklists should be tailored to specific surgical disciplines, institutions, geographic regions and countries [e.g, 13,15]. For example, some healthcare institutions have policies to reduce surgical risk specifically for patients undergoing orthopaedic surgery [17–25].

To determine where surgical safety initiatives need to be modified or focused for relevance to orthopaedic surgery, the incidence and predictors of adverse events must be identified. At this time, however, there is relatively little empirical research on the array of adverse events across the varied types of orthopaedic interventions. Most research focuses on the risk of adverse events following total knee and total hip replacement.

For example, Soohoo et al. [26] found that, following total knee replacement, the 90-day risk was 0.53% for mortality, 0.71% for serious infection, and 0.41% for pulmonary embolism [26]. They also reported that the overall 90-day risk of a complication (i.e., death, serious infection, pulmonary embolism etc) was positively associated with age, the Charlson Comorbidity Index, hospital volume, having private insurance, being male, and being white. In another study, Pulido et al. [27] reported 12-month infection risks following hip and knee arthroplasty of 0.3 and 1.1%, respectively. Stefansdottir et al. [28] suggested that such infections are closely related to inadequate timing of prophylactic antibiotics. In another study of adverse-event frequency for different orthopaedic procedures, Schilling et al. [29] found a 30-day mortality risk of 0.005% for all procedures. They found that hip fracture repair accounted for the greatest share of adverse events (19%), followed by total knee arthroplasty (18%), total hip arthroplasty (11%), and revision total hip arthroplasty (5%).

More recently, Browne et al. [30] found that across over 200,000 patients who underwent total joint arthroplasty, the following risks of in-hospital postoperative complications were found: anemia 16%, cardiac 0.45%, peripheral vascular 0.1%, respiratory 0.5%, gastrointestinal 0.3%, genitourinary 0.35%, central nervous system 0.1%, hematoma/seroma 0.8%, wound dihescense 0.04%, infection 0.15%, deep vein thrombosis 0.2%, pulmonary embolism 0.1% and mortality <0.1%. They also found that Medicaid patients had a higher risk for some of these complications.

While these studies are informative, we could not find any research that comprehensively explored the risk of adverse events across a wide array of orthopaedic procedures. They do not provide a detailed description of all types of adverse events in these patients, such that we can reliably and comprehensively inform safety initiative development. The objectives of this research were to extract data on a broad range of adverse events following orthopaedic surgeries in a hospital population, to estimate and compare the incidence rates and risks of those adverse events, and to assess associations of adverse events with selected patient factors and type of surgery. This information will help inform specific safety system development for this population of patients and in orthopaedic surgery.

## Methods

### Study design

We conducted a retrospective cohort study by manually reviewing and extracting from electronic medical records (EMR) data on the incidence and predictors of adverse events that occurred within 90 days for the 50 most frequent orthopaedic surgeries performed at the University of Michigan Health System in 2010. In this project, an adverse event was any negative patient outcome that occurred within 90 days of the patient having surgery and that was described by the investigators *a priori* or during data collection as possible consequences of surgery or hospitalization.

### Data source

All EMRs were accessed through CareWeb, a web-based clinical patient record system developed for use by clinicians and clinical support staff [31]. We reviewed and extracted data from the orthopaedic clinical notes, notification notes, phone notes, imaging documents and reports.

### Inclusion and exclusion criteria

We included patients who had an orthopaedic surgical procedure in 2010 for one of the 50 most common Current Procedural Terminology (CPT) codes (See Appendix 1). Patients were excluded if their surgery was a repeat procedure. Patients of any age and with any comorbidites were included.

### Data extraction

One individual (PK) extracted all data into preformatted excel spreadsheets for all included patients. A random sample of approximately ten percent of the first 100 patients was separately and independently reviewed by a second individual (JG) and a third individual, an orthopaedic surgeon, if needed. These individuals then met to discuss any discrepancies in the extraction through discussion and further review of the source EMRs.

First, we extracted demographic information from each patient’s EMR including age, sex, primary diagnoses related to the surgery (ICD-9 codes), specific type of surgery (CPT code), comorbidities (ICD-9 codes), and surgeon identification number. Patient names and surgeon names were coded by the data extractor and sent to the investigators to keep them blinded. The codes were kept in a secure location by the data extractor and only referred to by that person when additional data were required.

Adverse events were defined broadly as any of the following: an unintended injury, complication, prolonged hospital stay (greater than 30 days), disability observed at the time of discharge, or death. Unintended injuries or complications included repeat/revision surgery (i.e., due to wrong site surgery, long-term bleeding, non-healing of wound), surgical site infection, deep vein thrombosis, and the prolonged pain (i.e., 42 days or more of narcotic medication). Appendix 2 lists the adverse events and their definitions used during the data extraction process. We also considered events not predetermined and that were unanticipated but still determined to be possible adverse events by the data extractors (See Appendix 3 for other adverse events). The number of days after the orthopaedic surgical procedure corresponding to each adverse event was also extracted.

Patients were scored on two comorbidity indexes: the Charlson Comorbidity Index (CCI) [32], which was based on the prediction of mortality; and the Functional Comorbidity Index (FCI) [33], which was based on the prediction of functional status. The CCI contains patient data on 19 chronic conditions: acquired immune deficiency syndrome, myocardial infarction, congestive heart failure, peripheral vascular disease, dementia, chronic pulmonary disease, connective tissue disease, peptic ulcer disease, leukemia, lymphoma, tumor without metastasis, metastatic solid tumor, moderate or severe renal disease, cerebrovascular disease, liver disease, and diabetes. Each condition is assigned a weight (an integer from 1 to 6) based on the adjusted association between that condition and the mortality rate in one year (reported by Charlson et al. [32]), and the index score is the sum of the weights for all conditions reported (see Table 3 in Charlson et al. [32]). There is evidence that the CCI predicts outcomes following orthopaedic procedures (e.g., SooHoo et al. [26]).

The FCI was developed by Groll et al. [33] for use in general populations. They derived the FCI from self-reported diagnoses of 18 chronic conditions; the selected conditions predict the physical function subscale (10 items) of the medical outcomes study short form-36 (MOS SF-36). The FCI includes arthritis (osteoarthritis and rheumatoid), osteoporosis, asthma, angina, neurological disease, depression, anxiety or panic disorders, visual impairment, hearing impairment, degenerative disc disease, obesity (body-mass index > 30), chronic obstructive pulmonary disease, congestive heart failure, heart attack, stroke or transient ischemic attack, peripheral vascular disease, diabetes (type I and II), and upper gastrointestinal disease (see Table 5 in Groll et al. [33]). We used one of the recommended methods for scoring the FCI—a simple count of the number of conditions reported by each subject [33]. The FCI includes an important functional assessment that goes beyond the CCI and was developed in patients with musculoskeletal conditions and therefore is appropriate in our population.

### Statistical analysis

First, we described the demographic, surgical, and comorbidity characteristics of patients in the study population. For each type of adverse event (except prolonged pain), we estimated the incidence of that outcome in three ways: the rate of adverse events within the 90-day follow-up period (number of outcome events, divided by total person-time a risk), the 90-day risk (probability of experiencing at least one outcome event during follow-up); and the mean number of adverse events per procedure. We also calculated the mean number of days from surgery to the first occurrence of each type of event, and we examined the distribution of the number of adverse events per patient (including and excluding prolonged pain). For prolonged pain, we estimated the prevalence at 42 days. Patients lost to follow-up were censored at their last visit.

We used two methods to model the effects of selected variables on the risk or incidence rate of adverse events occurring within 90 days of surgery and the prevalence ratio of prolonged pain at 42 days. In all models, surgeons were treated as random effects using generalized estimating equations. First, modified Poisson regression was used to estimate the crude and adjusted risk ratios (RR) for the estimated effects of age, sex, and the CCI or FCI on adverse events within 90 days of surgery; or the prevalence ratio for prolonged pain at 42 days post-surgery. This method was also used to estimate the effects of early adverse events and specific comorbidities on prolonged pain. Second, negative binomial regression was used to estimate the crude and adjusted incidence rate ratios (IRRs) for the effects of age, gender, and the CCI or FCI (and individual comorbidities) on all adverse events occurring in patients during the 90-day follow-up period (i.e., counting all adverse events that occurred in each patient). We analyzed data in all models separately for events related to pain and for those undergoing TKA and THA. We adjusted for follow-up time in all models.

## Results

**Fig. 1:**
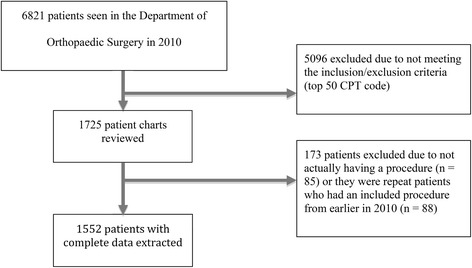
Patient inclusion flow chart

A total of 6,821 patients were seen in the Department of Orthopaedic Surgery in 2010, of which 1,552 were eligible for this study (see Fig. 1). The average age of all included patients was 53.4 years (range, 2–102); 51.7% were female. A total of 1,148 adverse events were identified in 729 patients. The mean length of hospital stay was 3.3 days (range 0–62). The proportion of all 1,552 patients that were not followed for at least 90 days after surgery was 34.9% (*n* = 541); of those not followed for at least 90 days, the mean duration of follow-up was 42.4 days. The rate of adverse events (number of events per total follow-up time) was 0.01 events per day, or 10 events per 1000 days.

**Fig. 2:**
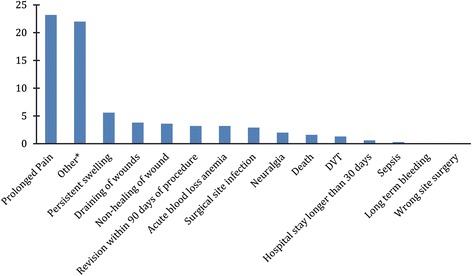
Ninety-day risk of adverse events across all included patients. *Other adverse events were identified by data extractors during chart review that were not predefined by the investigator. Examples of other adverse events include: urinary tract infection, urinary reten-tion, foot drop, fever, hematoma, pneumonia, bronchitis, myocardial infarction, allergic reaction to medication, hemarthrosis, venous thrombosis, significant blood loss with staple removal, heterotopic ossification

Figure 2 shows the 90-day risk of each type of adverse event. The number, proportion and estimated 90-day risk of each type of adverse event as well as the mean number of days post-op for those events among all patients is shown in Table 1. A total of 47% of all patients experienced at least one adverse event. The most frequent event was prolonged pain, representing 31% of all adverse events in the study; the 42-day prevalence was 23%. Sepsis, long-term bleeding, and wrong-site surgery had the lowest risks. There was a large number of rare adverse events (31% of the total) that we grouped together as “other” (see Appendix 2 for a complete list of “other” AEs). Aside from acute loss of blood, most adverse events tended to occur after hospital discharge.

**Table 1:** Adverse events across all patients

Type of Adverse Event	Number of Events (percent of total)	90-Day Event Risk (%)	Mean Days from Surgery to Event
Prolonged pain^a^	360 (31.36)	23.19	---
Persistent swelling	86 (7.40)	5.54	50.12
Draining of wounds	56 (4.87)	3.73	24.78
Non-healing of wound	56 (4.87)	3.61	30.38
Revision within 90 days of procedure	50 (4.36)	3.22	40.96
Acute blood loss anemia	50 (4.36)	3.22	1.92
Surgical site infection	44 (3.83)	2.84	27.09
Neuralgia	32 (2.78)	2.06	56.22
Death	25 (2.18)	1.61	30.44
DVT	21 (1.83)	1.35	21.95
Hospital stay longer than 30 days	10 (0.87)	0.64	41.70
Sepsis	4 (0.35)	0.26	24.00
Long term bleeding	2 (0.17)	0.13	13.00
Wrong site surgery	0	0	0
Other^b^	362 (31.53)	23.32	17.47

^a^Prolonged pain was defined as pain requiring narcotics for greater than 42 days or clearly stated in the patient’s chart as prolonged pain

^b^Examples of other adverse events: urinary tract infection, urinary retention, foot drop, fever, hematoma, pneumonia, bronchitis, myocardial infarction, allergic reaction to medication, hemarthrosis, venous thrombosis, significant blood loss with staple removal, heterotopic ossification

For procedures done at least 15 times in 2010, Table 2 describes the adverse events. Total hip and knee arthroplasty (THA, TKA) were the dominant procedures in this sample. A total of 53% of all adverse events in the study were in those patients who underwent THA, TKA, or arthrodesis in the lumbar spine. The mean number of AEs per procedure varied appreciably among procedures; it was highest (>1 AE/procedure) for application of external fixation system (1.48), lumbar arthrodesis (1.41), open treatment of a femur fracture (1.20), and debridement (1.18).

**Table 2:** Frequency of procedures and adverse events by type of procedure^a^

Procedure Type	Number of Procedures	Percent of all Study Procedures	Total Number of Adverse Events	Percent of all Adverse Events	Mean Number of Events per Procedure
Total hip arthroplasty	313	20.17	179	15.59	0.57
Total knee arthroplasty	252	16.24	243	21.17	0.96
Lumbar arthrodesis posterior - single	132	8.51	186	16.20	1.41
Removal of deep implant	132	8.51	79	6.88	0.60
Open treatment of femur fracture & interlocking nail	65	4.19	78	6.79	1.20
Arthroscopy of the knee and partial menisectomy	64	4.12	18	1.57	0.28
Debridement: skin, tissue, muscle, bone	60	3.87	71	6.18	1.18
Anterior cruciate ligament repair with arthroscopy and autograft	53	3.41	11	0.96	0.21
Open treatment femoral fracture proximal end/neck with fixation	53	3.41	43	3.75	0.81
Neurolysis median nerve at carpal T	38	2.45	12	1.05	0.32
Application of external fixation system, unilateral; uniplanar	31	2.00	46	4.01	1.48
Slipped capital femoral epiphysis with femoral neck osteoplasty	31	2.00	14	1.22	0.45
Arthroscopy of shoulder, surgical; with rotator cuff repair	32	2.06	12	1.05	0.38
Open treatment and fixation of the clavicle	29	1.87	14	1.22	0.48
Gastronemius recession	29	1.87	26	2.26	0.90
Excisional bone biopsy - deep	25	1.61	12	1.05	0.48
Arthroscopy of the shoulder, surgical; capsulor repair	24	1.55	6	0.52	0.25
Arthroscopy of the shoulder with acromioplasty	22	1.42	8	0.70	0.36
Arthroscopy, debridge, drill, resect	17	1.10	7	0.61	0.41
Removal of exterior fixator system	16	1.04	12	1.05	0.75
Open toe flexor tenotomy, single	15	0.97	8	0.70	0.53
Other study procedures	130	8.38	82	0.07	0.78
Total study procedures	1552	100	1148	100	0.73

^a^This table lists only those procedures that were done 15 or more times

The adverse events for the top two procedures, THA and TKA are listed in Table 3. For THA other and prolonged pain were by far the most common adverse events (41.9 and 33% of the total events), followed by draining of wounds (7.3%). Those undergoing THA did not have any of the following events: hospital stay longer than 30 days, wrong site surgery, long term bleeding, or sepsis. For TKA patients, the most frequent adverse events were prolonged pain (32.1%) followed by other events (31.7%) and then persistent swelling (12.8%), with no patients having a hospital stay longer than 30 days, wrong site surgery, long term bleeding, or sepsis.

**Table 3:** Adverse events for total hip or total knee arthroplasty only

Adverse Event	Total Hip Arthroplasty, *N* = 313 # (%)	Total Knee Arthroplasty, *N* = 252 # (%)	Total
Death	1 (0.6)	1 (0.4)	2
Hospital stay longer than 30 days	0	0	0
Revision within 90 days of procedure	7 (3.9)	21 (8.6%)	28
Wrong site surgery	0	0	0
Long term bleeding	0	1 (0.4)	1
Non-healing of wound	4 (2.2)	12 (4.9)	16
Persistent swelling	2 (1.1)	31 (12.8)	33
Surgical site infection	7 (3.9)	6 (2.5)	13
Sepsis	0	0	0
Draining of wounds	13 (7.3)	4 (1.6)	17
DVT	1 (0.6)	1 (0.4)	2
Prolonged Pain	59 (33.0)	78 (32.1)	137
Nerve Pain	4 (2.2)	2 (0.8)	6
Acute Blood Loss Anemia	7 (3.9)	8 (3.3)	15
Other	75 (41.9)	77 (31.7)	152
Totals	179	243	422

Table 4 shows the number and percentage of patients by the number of adverse events per patient, including and excluding prolonged pain. The percentage of all study patients who experienced more than one adverse event during the 90-day follow-up period was 16% when including prolonged pain and 11% when excluding prolonged pain.

**Table 4:** Proportion of patients with multiple adverse events

Number of Adverse Events	Including Pain		Excluding Pain	
	Total Number of Patients	Percentage	Total Number of Patients	Percentage
0	828	53.01	1050	67.22
1	470	30.09	338	21.64
2	160	10.24	101	6.47
3	63	4.03	42	2.69
4	24	1.54	20	1.28
5	11	0.70	7	0.45
6	5	0.32	4	0.26
7	1	0.06	0	0

Crude and adjusted incidence rate ratios (IRR) from the negative binomial regression analyses for the rate of adverse events (excluding prolonged pain) are shown in Table 5, separately for all study procedures (Panel A; *N* = 1192) and for THA and TKA only (Panel B; *N* = 413). More comorbidity, as measured by both the CCI and FCI scores, was positively associated with the rate of adverse events in the adjusted analyses involving all procedures, but the association was stronger for FCI than for the CCI (adjusted IRR for FCI = 1.34; 95% CI: 1.23 to1.46; adjusted IRR for CCI = 1.20; 95% CI: 1.11 to 1.31). Both age and sex were unassociated with the rate of adverse events. When restricting the analysis to only TKA and THA procedures, age was positively but weakly associated with the AE rate in both model 1, adjusting for sex and CCI (adjusted IRR per 10 years = 1.15; 95% CI: 1.00 to 1.32) and model 2, adjusting for sex and FCI (adjusted IRR per 10 years = 1.12; 95% CI: 0.99 to 1.29).

**Table 5:** Crude and adjusted associations for age/10, sex, CCI and FCI with the number of adverse events (excluding pain) across all procedures (Panel A) and for only THA and TKA procedures (Panel B) using negative binomial regression modeling

Predictors	Incidence Rate Ratio	95% CI	*p*-value
A. All Procedures (*N* = 1192)			
Crude Associations			
Age (decade)	1.00	0.95–1.06	0.87
Sex (male coded as 1)	0.99	0.81–1.21	0.91
CCI score (per 1 point)	1.20	1.11–1.31	<0.001
FCI score (per 1 point)	1.34	1.23–1.45	<0.001
Adjusted Models			
*Model 1*			
Age (decade)	1.00	0.95–1.06	0.92
Sex	0.99	0.81–1.21	0.95
CCI score (per 1 point)	1.20	1.11–1.31	<0.001
*Model 2*			
Age (decade)	0.99	0.94–1.04	0.67
Sex	0.99	0.81–1.22	0.97
FCI score (per 1 point)	1.34	1.23–1.46	<0.001
B. THA and TKA Procedures Only (*N* = 413)			
Crude Associations			
Age (decade)	1.16	1.01–1.33	0.03
Sex	1.15	0.82–1.62	0.41
CCI score (per 1 point)	1.20	1.04–1.39	0.02
FCI score (per 1 point)	1.24	1.09–1.41	0.001
Adjusted Models			
*Model 1*			
Age (decade)	1.15	1.00–1.32	0.04
Sex	1.13	0.80–1.60	0.47
CCI score (per 1 point)	1.18	1.02–1.37	0.03
*Model 2*			
Age (decade)	1.12	0.99–1.29	0.08
Sex	1.16	0.82–1.64	0.40
FCI score (per 1 point)	1.22	1.07–1.39	<0.001

*CCI* Charlson comorbidity index, *FCI* functional comorbidity index, *THA* total hip arthroplasty, *TKA* total knee arthroplasty

Table 6 shows the crude and adjusted associations of age, sex, and CCI or FCI with the 90-day risk of any adverse event (excluding prolonged pain), using modified Poisson regression, for all study procedures (Panel A) and for THA and TKA procedures only (Panel B). In the adjusted analyses for all procedures, increasing FCI and CCI scores were associated with a small increased risk of any AE (adjusted RR for 1 point on the CCI scale = 1.10; 95% CI: 1.02 to 1.18; and adjusted RR for 1 point on the FCI scale = 1.19, 95% CI: 1.13 to 1.25). Age and sex were minimally associated with the risk of AEs. In the adjusted analysis including only those patients undergoing THA or TKA, men were more likely than women to experience an AE within 90 days (RR adjusting for age and CCI = 1.22; 95% CI: 0.98 to 1.51; RR adjusting for age and FCI = 1.23; 95% CI: 1.01 to 1.51). In these restricted analyses, the comorbidity scores were not as strongly associated with AEs as in the analyses of all procedures (adjusted RR for 1 point on the CCI scale = 1.07; 95% CI: 0.99 to 1.15; and adjusted RR for 1 point on the FCI scale = 1.13; 95% CI: 1.06 to 1.21).

**Table 6:** Crude and adjusted associations for age/10, sex, CCI and FCI with the 90-day risk of an adverse event (excluding pain) across all procedures (Panel A) and for only THA and TKA procedures (Panel B) using modified Poisson regression modeling

Predictors	Risk Ratio	95% CI	*p*-value
Panel A: All Procedures (*N* = 1192)			
Crude Associations			
Age (decade)	1.03	0.99–1.07	0.12
Sex	1.02	0.81–1.29	0.85
CCI score (per 1 point)	1.10	1.02–1.18	0.01
FCI score (per 1 point)	1.19	1.13–1.25	<0.001
Adjusted Models			
*Model 1*			
Age (decade)	1.03	0.99–1.07	0.13
Sex	1.04	0.82–1.30	0.76
CCI score (per 1 point)	1.10	1.01–1.18	0.02
*Model 2*			
Age (decade)	1.02	0.98–1.06	0.28
Sex	1.03	0.82–1.30	0.80
FCI score (per 1 point)	1.19	1.13–1.25	<0.001
Panel B: THA and TKA Procedures Only (*N* = 413)			
Crude Associations			
Age (decade)	1.12	0.96–1.31	0.15
Sex	1.23	1.01–1.50	0.04
CCI score (per 1 point)	1.09	0.98–1.20	0.104
FCI score (per 1 point)	1.14	1.07–1.22	<0.001
Adjusted Models			
*Model 1*			
Age (decade)	1.12	0.97–1.28	0.12
Sex	1.22	0.98–1.51	0.07
CCI score (per 1 point)	1.07	0.99–1.15	0.07
*Model 2*			
Age (decade)	1.11	0.96–1.27	0.16
Sex	1.23	1.01–1.51	0.04
FCI score (per 1 point)	1.13	1.06–1.21	<0.001

1. For the variable sex, female was coded as a 0 and male as a 1

*CCI* Charlson comorbidity index, *FCI* functional comorbidity index, *THA* total hip arthroplasty, *TKA* total knee arthroplasty

Table 7 shows the crude and adjusted prevalence ratios for the associations of age, sex, and CCI or FCI with the 42-day prevalence of prolonged pain, using modified Poisson regression, for all procedures and for THA and TKA procedures only. In the analysis of all procedures, the FCI score was positively associated with the prevalence of prolonged pain at 42 days (adjusted prevalence ratio (PR) = 1.10; 95% CI: 1.01 to 1.19). Age, sex, and CCI score were only minimally associated with the prolonged pain. In contrast, these results were different for those analyses restricted to THA and TKA procedures. Men were 42% more likely than women to report prolonged pain at 42 days (PR adjusting for age, sex, and CCI = 1.42; 95% CI: 1.09 to 1.86; and PR adjusting for age, sex, and FCI = 1.42; 95% CI: 1.10–1.85). There was little association between age and prolonged pain, and the associations between the comorbidity scores and prolonged pain were weaker than observed for other AEs (adjusted PR for 1 point on the CCI scale = 1.02; 95% CI: 0.84 to 1.25; and adjusted PR for 1 point on the FCI scale = 1.06; 95% CI: 1.01 to 1.13).

**Table 7:** Crude and adjusted associations for age/10, sex, CCI and FCI with the adverse event of pain or not at 42 days across all procedures (Panel A) and for only THA and TKA procedures (Panel B) using modified Poisson regression modeling

Predictors	Prevalence Ratio	95% CI	*p*-value
Panel A: All Procedures (*N* = 1552)			
Crude Associations			
Age (decade)	1.02	0.97–1.08	0.36
Sex	0.85	0.68–1.05	0.13
CCI score (per 1 point)	1.02	0.88–1.19	0.77
FCI score (per 1 point)	1.10	1.02–1.20	0.02
Adjusted Models			
*Model 1*			
Age (decade)	1.02	0.97–1.08	0.38
Sex	0.85	0.68–1.05	0.14
CCI score (per 1 point)	1.02	0.87–1.20	0.81
*Model 2*			
Age (decade)	1.02	0.97–1.07	0.46
Sex	0.85	0.68–1.06	0.16
FCI score (per 1 point)	1.10	1.01–1.19	0.03
Panel B THA and TKA Procedures Only (*N* = 550)			
Crude Associations			
Age (decade)	0.98	0.92–1.05	0.57
Sex	1.42	1.10–1.85	0.01
CCI score (per 1 point)	1.03	0.86–1.24	0.76
FCI score (per 1 point)	1.06	1.01–1.12	0.03
Adjusted Models			
*Model 1*			
Age (decade)	0.98	0.91–1.06	0.68
Sex	1.42	1.09–1.86	0.01
CCI score (per 1 point)	1.02	0.84–1.25	0.82
*Model 2*			
Age (decade)	0.98	0.91–1.05	0.56
Sex	1.42	1.10–1.85	0.01
FCI score (per 1 point)	1.06	1.01–1.13	0.03

1. For the variable sex, female was coded as a 0 and male as a 1

*CCI* Charlson comorbidity index, *FCI* functional comorbidity index, *THA* total hip arthroplasty, *TKA* total knee arthroplasty

Using modified Poisson regression, we also examined associations between specific AEs occurring within 42 days of surgery and the prevalence of prolonged pain at 42 days, but the numbers were too small to adjust for covariates. Nevertheless, we found a strong crude association between surgical site infection and prolonged pain (crude PR = 3.59; 95% CI: 2.22 to 5.80; *p* < 0.001).

Using negative binomial regression, we also estimated the effects of specific comorbidities on the incidence rate of all AEs (excluding prolonged pain). We found positive associations, adjusting for age, sex and duration of follow-up, for essential hypertension (IRR = 1.45; 95% CI: 1.16 to 1.83), asthma (IRR = 1.80; 95% CI: 1.08 to 3.02), and chronic airway obstruction (IRR = 1.81; 95% CI: 1.18 to 2.78). Finally, we examined the relation of asthma and essential hypertension with selected AEs, adjusting for age and sex, using modified Poisson regression. We found that asthma was strongly associated with the prevalence of prolonged pain at 42 days (PR = 13.3; 95% CI: 8.84 to 20.0), and it was inversely associated with acute blood loss (RR = 0.52; 95% CI: 0.32 to 0.84).

## Discussion

We found that in patients undergoing the 50 most common orthopaedic procedures at the University of Michigan in 2010, the 90-day risk of having an adverse event after surgery was nearly 50%. These findings are consistent with those of previous studies.

For example, Ouchterlony et al. [34] found that in a sample of 1,361 patients undergoing general, vascular, or orthopaedic surgery, AEs were noted in 47% of patients in the postoperative unit. In another study of 125,000 Medicare beneficiaries who had undergone unilateral primary TKA and another 11,726 for revision TKA, Mahomed et al. [35] found that within 90 days of the procedure 5.0% of primary and 10.2% of revision patients had an AE, as identified through searching for ICD-9 claims codes. These risks are appreciably lower than our estimates (TKA 90-day risk of 21%), and this discrepancy likely derives from different definitions of AEs, different methods of identifying the AEs, and different patients populations. In another study, Wolf et al. [36] looked at Medicare beneficiaries who had undergone primary THA (*N* = 1,405,379) and revision THA (*N* = 337,874) between 1991 and 2008. They found a 90-day risk of AEs in the primary THA group of 3.4 to 4.0% across the study period and for the revision THA group 7.0 to 10.9% across the study period. Again, these risks were much lower than what we found in our study (THA 90-day risk of 16%), likely resulting from the differences in definitions of AEs, data extraction methods, and patient populations. Furthermore, we were able to characterize the proportions of specific adverse events in those undergoing THA or TKA (see Table 3), which expands the literature in this area. These latter findings may help orthopaedic surgeons monitor and potentially prevent adverse events in those undergoing total joint arthroplasty.

We found that prolonged pain was by far the most frequent outcome, with a 42-day prevalence of 23% across all procedures and 28.7% in patients undergoing THA and 20.0% in patients undergoing TKA. Other researchers have looked at pain in patients undergoing total joint arthroplasty. For example, in one study of THA and TKA patients, Carroll et al. [37]) found that the median time to pain resolution was 81 days (95% CI: 49 to 146 days) in TKA patients and 81 days (95% CI: 43 to 146 days) in THA patients. While the outcome used in their study is different than ours, their findings indicate that more than 50% of patients in their sample would have had prolonged pain as defined in our project, because the median time to pain resolution was greater than 42 days in both THA and TKA patients. The higher proportion of patients with prolonged pain is not surprising, given that they defined pain differently. Carroll et al. defined pain as any type of pain included in the Brief Pain Inventory (BPI), which includes a variety of questions on the person’s perception of pain.

We found that the mean number adverse events was greatest for lumbar spinal surgery and fixation of an external fixation system. These findings are not surprising, given that these procedures are considerably invasive, the latter of which resulting from trauma. Other investigators have found a very similar frequency of adverse events following spinal surgery (e.g., Hellsten et al. [38]) and that trauma patients have higher risks of complications [39]. For example, a recent analysis of National Surgical Quality Improvement Program (NSQIP) data on 146,773 orthopaedic patients (22,361 trauma patients) found that trauma patients had a higher risk of complications [39].

Across all of our adjusted regression models the FCI positively predicted the incidence of all adverse events, and the strength of that association was consistently greater than for the CCI. Given that the FCI was developed to predict function, its predictive capability for AEs in this population of orthopaedic patients was expected. To our knowledge, our study is the first to show the association between the FCI and adverse events following orthopaedic surgery. In our models for THA and TKA only, being older predicted an increased rate of AEs and being male increased the risk of any AE. Other research in patients undergoing revision THA and TKA showed an increased risk for AEs in older patients and in men [35, 40].

In our adjusted regression models, we found that essential hypertension, asthma and chronic airway obstruction were positively associated with the rate of all AEs within 90 days of an orthopaedic procedure. When we examined these relations further, we found that asthma was strongly and positively associated with the 42-day prevalence of prolonged pain and inversely associated with the risk of acute blood loss. The relation between individual comorbidities and AEs has been explored in other research. For example, Minhas et al. [41], using data from the NSQIP database on 42,150 patients, explored the relation of a number of variables on the occurrence of a cerebral vascular accident (CVA) following an orthopaedic procedure and reported in their adjusted analysis that hypertension, dyspnea and chronic obstructive pulmonary disease (COPD) were strongly associated with CVAs. But they also found that insulin dependent diabetes mellitus and a history of a transient ischemic attack predicted CVAs as well. While we found that diabetes mellitus predicted individual and multiple AEs in our crude models, when adjusted for age sex and other comorbidities, the relation was eliminated. Of course, in our study we looked across all AEs, not just CVAs. Other research on the risk of AEs in TKA patients while hospitalized, also failed to show an association between diabetes and the risk of AEs, but did find that COPD strongly predicted the risk of AEs [42]. To the best of our knowledge, the relation we found between asthma and the 42-day prevalence of prolonged pain has not been reported elsewhere. Future rigorous research is required to confirm this finding.

Our study has several strengths. We included a sample of all patients undergoing the top 50 orthopaedic procedures at a large academic center. Therefore, we expect our findings to be generalizable to orthopaedic patients undergoing the same procedures at other academic centers. We extracted a large amount of data from patient charts and considered the most practical approach for identifying AEs in a large number of patients following surgical procedures [43]. Furthermore, we were careful to be certain that extractions were cross-checked. Thus, we are confident that our data extraction was complete and reliable. In addition, we extracted a large selection of potential AEs, which goes beyond what has been done in other research. Furthermore, we performed many careful analyses, attempting to delineate associations so as to inform further research in the area.

One potential drawback of our study is that the generalizability does not necessarily extend beyond academic centers or to procedures not included in our analyses. Furthermore, it is possible that the charts in the EMR for each patient may suffer from unclear or underreporting of AEs (reporting bias), resulting in biased estimates of rates and risks in this study. For example, it is possible that certain procedures or conditions may cause attending physicians to look harder for potential adverse events or chart them more frequently for those patients, resulting in detection bias. The role of reporting bias and detection biases as it relates to chart reviews of AEs should be explored further. Another potential drawback of our study is that almost 35% of the included patients had less than 90 days of follow-up and a mean follow-up of approximately 42 days. The lack of complete follow-up in these patients could have biased our findings; thus, caution is advised when interpreting our findings. Finally, because we did not compare the incidence of outcome events in surgical vs. non-surgical patients, we cannot make any inferences about the effects of surgery or hospitalization on the occurrence of adverse events in this study; i.e., the adverse events were not necessarily caused by the patients’ surgery or hospitalization that preceded them.

## Conclusion

We found that patients undergoing orthopaedic procedures are likely to experience a broad spectrum of adverse events. Some of those events have serious implications to the patient’s health, whereas many others are temporary or can easily be treated. Indeed, the types of AEs considered in this study are broader than the types of events described in previous studies. We also found that certain patient characteristics, especially comorbidities, are associated with the incidence of adverse events. These findings may aid clinicians in identifying which patients are at an increased risk for certain AEs. We feel that these findings support the need for large prospective (possibly randomized) studies, with careful patient follow-up to delineate the risk of particular AEs and to assess which AEs are in fact affected by orthopaedic procedures.

## Appendix 1

**Table 8:** CPT Code Descriptions and Volume

CPT Code	Procedure Description	Volume
20610	ASPIRATE OR INJECT MAJOR JOINT 13	5
20610	ASPIRATE OR INJECT MAJOR JOINT	3,704
20610	ASPIRATE/INJECT MAJOR JOINT	14
20610 Total		3,723
20680	REMOVAL OF IMPLANT - DEEP 13	178
20680	REMOVAL OF IMPLANT - DEEP	342
20680 Total		520
29881	ARTHROSCOPY,KNEE & PART MENISECTOMY	270
29881	ARTHROSCOPY,KNEE & PART MENISECTOMY	83
29881 Total		353
27130	TOTAL HIP ARTHROPLASTY	341
27130	TOTAL HIP REPLACE W CALCAR	6
27130 Total		347
29826	ARTHROSCOPY SHOULDER W ACROMIOPLASTY	184
29826	ARTHROSCOPY SHOULDER W/ACROMIOPLASTY	108
29826 Total		292
27447	TOT KNEE ARTHROPLASTY-MED & LATERAL	281
27447	TOT KNEE ARTHROPLASTY-MED & LATERAL 13	5
27447 Total		286
29888	ARTHROSCOPIC ACL RECONSTRUCTION	4
29888	ARTHROSCOPIC ACL RECONSTRUCTION	32
29888	ACL REPAIR W ARTHROSCOPE AND AUTOGRAFT 13	1
29888	ACL REPAIR W ARTHROSCOPE AND AUTOGRAFT	215
29888 Total		252
29822	ARTHROSCOPY,SHOULDER,SURGICAL;LIMIT 13	223
29822	ARTHROSCOPY,SHOULDER,SURGICAL;LIMIT	7
29822 Total		230
20605	ASPIRATE OR INJECT INTERMED JOINT 13	3
20605	ASPIRATE OR INJECT INTERMED JOINT	205
20605 Total		208
22614	FUSION POSTR EA ADD SEG	193
22614 Total		193
29827	ARTHROSCOPY,SHOULDER,SURGICL; W ROTATR CUFF REPAIR	170
29827	ARTHROSCOPY,SHOULDR,SURGICL;W ROTATR CUFF REPAIR	3
29827 Total		173
29877	ARTHROSCOPY,DEBRIDGE,DRILL, RESECT	120
29877	ARTHROSCOPY,DEBRIDGE,DRILL,RESECT	52
29877 Total		172
29450	APPLICA CLUBFOOT CAST UNILATERAL	159
29450	APPLIC CLUBFOOT CAST UNILATERAL	2
29450	APPLICA CLUBFOOT CAST UNILATERAL 13	1
29450 Total		162
20936	AUTOGRAFT FOR SPINE SURG ONLY,LOC THRU SAME INCIS;LIST SEP	160
20936 Total		160
64721	NEUROLYSIS MEDIAN NERVE AT CARPAL T 13	17
64721	NEUROLYSIS MEDIAN NERVE AT CARPAL T	139
64721 Total		156
26055	TENDON SHEATH INCISION-TRIGGER FING	100
26055	TENDON SHEATH INCISION-TRIGGER FING 13	41
26055 Total		141
20930	MORSELIZED ALLOGRAFT, FOR SPINE SURGRY;LIST SEP IN ADDITN	109
20930 Total		109
22843	POSTR INSTRU 7 - 12 SEGMENTS; LIST SEPAR	109
22843 Total		109
11012	DEBRIDE: SKIN, TISSUE, MUSCLE, BONE 13	29
11012	DEBRIDE: SKIN, TISSUE, MUSCLE, BONE 13	75
11012 Total		104
22849	REINSERTION SPINAL FIXATION DEVICE	68
22849	REINSERTION SPINAL FIXATION DEVICE	36
22849 Total		104
25500	TX RADIUS SHAFT FRACTURE W/O MANIPU 13	1
25500	TX RADIUS SHAFT FRACTURE W/O MANIPU	102
25500 Total		103
22612	ARTHRODESIS POSTERIOR LUMBAR-SINGLE	99
22612 Total		99
29405	APPLICATION OF SHORT LEG CAST	73
29405	APPLICATION/SHORT LEG CAST	25
29405 Total		98
29875	ARTHROSCOPY & PLICA&/OR SHELF RESECT	32
29875	ARTHROSCOPY &PLICA&/OR SHELF RESECT	66
29875 Total		98
29806	ARTHROSCOPY,SHOULDER,SURG,CAPSULORRH	2
29806	ARTHROSCOPY,SHOULDER,SURG; CAPSULORRH	94
29806 Total		96
20550	INJECT SINGL TENDON SHEATH OR LIGAMENT	85
20550	INJECT SINGL TENDON SHEATH OR LIGAM	9
20550 Total		94
22842	POSTERIOR INSTRUMENTATION-SEPARATE LISTING	90
22842 Total		90
22802	ARTHRODESIS-7 OR MORE VERTEBRAE & GRAFT	86
22802	ARTHRODESIS-7 OR MORE VERTEBRAE W GRAFT	1
22802 Total		87
28232	OPEN TOE FLEXOR TENOTOMY SINGLE	6
28232	OPEN TOE FLEXOR TENOTOMY SINGLE 13	80
28232 Total		86
27179	OR SCFE W/FEMORAL NECK OSTEOPLASTY 13	49
27179	OR SCFE W/FEMORAL NECK OSTEOPLASTY	34
27179 Total		83
29075	APPLICATION SHORT ARM CAST (OSA)	41
29075	APPLICAT SHORT ARM CAST	2
29075	APPLICATION SHORT ARM CAST (OSA)	35
29075 Total		78
29874	ARTHROSCOPY KNEE AND REMOVAL OF BODY	66
29874	ARTHROSCOPY KNEE AND REMOVAL OF BODY	10
29874 Total		76
27245	OPEN TX FEMUR FX&INTERLOCKING NAIL 13	1
27245	OPEN TX FEMUR FX&INTERLOCKING NAIL	73
27245 Total		74
23440	RESECT OR TRANSPLANT BICEPS TENDON	8
23440	RESECT OR TRANSPLANT BICEPS TENDON 13	64
23440 Total		72
29862	ARTHROSCOPY HIP W/DEBRDMT ART CART	17
29862	ARTHROSCOPY HIP W DEBRDMT ART CART 13	55
29862 Total		72
24538	TX CLOSED SUPRA/TRANSCOND FX & FIXATION 13	1
24538	TX CLOSED SUPRA/TRANSCON FX & FIXATION	69
24538 Total		70
27193	CLOSED TRTMT PELVIC RQ FX; WO MANIPULATION	69
27193 Total		69
29882	ARTHROSCOPY,KNEE,SURGICAL & MEN REPAIR 13	33
29882	ARTHROSCOPY,KNEE,SURGICAL & MEN REPAIR	36
29882 Total		69
29880	KNEE ARTHROSCOPY W MED N LAT MENSTY	58
29880	KNEE ARTHROSCOPY W MED N LAT MENSTY 13	9
29880 Total		67
29425	APPLICAT SHORT LEG WALKING CAST	5
29425	APPLICATION SHORT LEG CAST WALKING	56
29425	APPLICATION SHORT LEG CAST WALKING	3
29425 Total		64
20694	REMOVAL EXT FIXATOR SYSTEM 13	38
20694	REMOVAL OF HOFFMAN DEVICE 13	6
20694	REM EXT FIXATOR IN CLINIC	1
20694	REMOVAL EXT FIXATOR SYSTEM	14
20694	REMOVAL OF HOFFMAN DEVICE	4
20694 Total		63
20690	APPLCATN EXTERNAL FIXATN SYSTEM, UNILAT; UNIPLANE	17
20690	APPLCATN EXTERNAL FIXATN SYSTEM UNIPLANE;UNILAT	24
20690	APPLICATION EXT FIX SYSTEM-UNIPLANE, UNILAT	13
20690	APPLICATION EXT FIX SYSTEM-UNILAT, UNIPLANE	8
20690 Total		62
22852	REMOVAL POSTERIOR INSTRUMENTATION	17
22852	REMOVAL POSTERIOR INSTRUMENTATION 13	43
22852 Total		60
29824	ARTHROSCPY,SHOULDER, SURGCL;W/DISTAL CLAV	3
29824	ARTHROSCPY,SHOULDER, SURGCL; W/DISTAL CLAV	56
29824 Total		59
63048	LAMINECTOMY, EA ADDTL SEGMENT 13	59
63048 Total		59
20245	EXCISIONAL BONE BIOPSY - DEEP (OSA) 13	46
20245	EXCISIONAL BONE BIOPSY - DEEP (OSA)	12
20245 Total		58
27236	OPEN TX FEM FX PROX END/NECK W/FIX	54
27236	OPEN TX FEM FX PROX END/NECK W/FIX 13	3
27236 Total		57
22310	TRTMT VERT BODY FX W/O MANIP	56
22310 Total		56
23515	OPEN TX FX CLAVICLE(INCL FIXATN WHEN PERF)	54
23515	OPEN TX FX CLAVICLE INCL FIXN WHEN PERFORMD	1
23515 Total		55
27687	GASTRONEMIUS RECESSION(EG,STRAYER)	37
27687	GASTRONEMIUS RECESSION(EG,STRAYER)	17
27687 Total		54
27792	OPEN TX LATERAL MALLEOLUS FX INCLD INT FIXN IF PERFORMED	49
27792	OPEN TX LATERAL MALLEOLUS FX INCL INTRN FIX WHEN PERFORMD	5

## Appendix 2

**Table 9:** Adverse event list and definitions/source

Adverse Event	Definition
Death	Reported in EHR
Hospital stay longer than 30 days	Reported in EHR
Revision within 90 days of procedure	Reported in EHR
Wrong site surgery	Reported as such in EHR
Long-term bleeding	Reported as such in EHR
Non-healing of wound	Reported as such in EHR
Persistent swelling	Reported as such in EHR
Surgical site infection	Reported as such in EHR
Sepsis	Reported as such in EHR
Persistent Draining of wounds	Reported as such in EHR
Deep Vein Thrombosis	Reported as such in EHR
Prolonged Pain	Greater than 42 days on narcotics or reported in EHR
Nerve Pain	Reported as such in EHR
Additional Medication Required	Reported as such in EHR
Acute Blood Loss/Anemia	Reported as such in EHR
Other^a^	Determined by review of physician of additional EHR reported events

^a^Musculoskeletal, non-union, urinary retention, urinary tract infection, gastrointestinal, foot drop, intra-operative complications, cranial/cerebral, pulmonary, re-injury, neuronal, hematoma, cardiac, reactions limb discrepancy, hematological, hematuria, integumentary, seromas, renal, chest pain

## Appendix 3

**Table 10:** Other Adverse Events

Event Category	Number	Mean Days Post-Op (95% CI)	Actual Events
Musculoskeletal	18	42.72 (12.94–29.78)	Bone ulcer, bursitis, post-op weakness, tendonitis, hemarthrosis, paresis, back spasms, tendon dysfunction,
Non-union of a fracture	1	64	N/A
Urinary retention	17	22.88 (0–46.70)	N/A
Gastrointestinal	35	15.03 (10.77–19.29)	Esophagitis, emphysematous gastritis, bloody stool, GI bleed, bowel obstruction, G tube infection, diarrhea, constipation, ileus, GI stress, C. Diff, acute colonic pseudo obstruction, continued nausea/vomit, acute on chronic vascular insufficiency of the bowel, PEG tube, Dobhoff tube falling out, viral gastroenteritis, dysphagea, dehydration, enema, colonic distention
Urinary tract infection	49	12.20 (8.46–15.95)	N/A
Foot drop	7	20.14 (2.61–37.68)	N/A
Intra-op complications	24	0	Seizure, severe bleeding after tornequet removed, partial avulsion fxs, Pt awake during surgery, Iatrogentic fxs, broken retained needle, vessels and arteries encountered, inferomedial neck fx, dural tear, retained drain tip, difficult intubation resulting in chipped tooth, tibial tubericle avulsion fx, peri-op NSTEMI, biceps tendon tear, ST elevation, failed sending of specimen from OR
Psychological/cranial/cerebral	24	11.38 (4.54–18.21)	disoriented, oversedation, syncopal episode, somnolence, delerium, double vision, non-convulsive status, altered mental status, TIAs, postop confusion, anoxic brain injury, gabapentin toxocity w/altered mental status, presyncope drug overdose, sleeping problems, withdrawl symptoms
Pulmonary	50	14.30 (10.71–17.89)	hypercarbia, O2 dependence, respiratory arrest, hypercarbic resp failure, pneumothorax, dyspnea, Pes
Re-injury	10	30.70 (12.82–48.58)	dislocations, screws backing out, re-fracturing, dural tears post-op
Neuronal	16	57.88 (45.22–70.53)	Nerve palsy, dysesthesia, numbness, weakness, paresis in BLE, mild dyskinesia, back spasms
Hematoma	13	14.77 (9.49–20.04)	N/A
Cardiac	29	5.90 (2.55–9.24)	Afib, hypotension, venous congestion, asymp tachycardia, desat, cardiac arrest, hypertension, angina, post-op hypotension, PEA bradycardia rhythm, junctional arrhythemia, ischemia, myocardial infarction, hypoxia
Allergic or treatment reactions	17	15.71 (7.37–24.04)	Allergic, blood transfusion, vomitting on narcs, anaphylaxis, staple reactions, increased INR
Limb-length discrepancy	3	44.67 (7.13–24.04)	N/A
Hematological	8	7.38 (3.38–11.37)	epistaxis, superficial VT, thrombocytosis, bacteremia, TIA, hyponatremia increased blood glucose, chronic vascular insufficiency
Hematuria	3	13 (0–33.01)	N/A
Integumentary	28	30.54 (22.44–38.63)	Suture sinus, furuncle, blood blister, fracture blister, retained sutures
Seromas	4	22.75 (4.89–40.61)	N/A
Renal	5	2.20 (0–4.38)	Acute on chronic renal failure, postop acute renal failure, renal failure
Chest Pain	2	44.50 (0–102.02)	N/A

## Abbreviations

AEs: Adverse events
CCI: Charlson comorbidity index
CI: Confidence interval
COPD: Chronic obstructive pulmonary disease
CPT: Current procedural terminology
CVA: Cerebral vascular accident
EMR: Electronic medical records
FCI: Functional comorbidity index
IRR: Incidence rate ratio
NSQIP: National Surgical Quality Improvement Program
PR: Prevalence ratio
THA: Total hip arthroplasty
TKA: Total knee arthroplasty
